# Recent Advances in Membranes Used for Nanofiltration to Remove Heavy Metals from Wastewater: A Review

**DOI:** 10.3390/membranes13070643

**Published:** 2023-07-04

**Authors:** Cristina Ileana Covaliu-Mierlă, Oana Păunescu, Horia Iovu

**Affiliations:** 1 Faculty of Biotechnical Systems Engineering, University Politehnica of Bucharest, 313 Splaiul Independentei, 060042 Bucharest, Romania; oana.stoian@upb.ro; 2 Advanced Polymer Materials Group, Faculty of Chemical Engineering and Biotechnologies, University Politehnica of Bucharest, 132 Calea Grivitei, 010737 Bucharest, Romania; horia.iovu@upb.ro

**Keywords:** membranes, nanofiltration, heavy metal, removal, wastewater

## Abstract

The presence of heavy metal ions in polluted wastewater represents a serious threat to human health, making proper disposal extremely important. The utilization of nanofiltration (NF) membranes has emerged as one of the most effective methods of heavy metal ion removal from wastewater due to their efficient operation, adaptable design, and affordability. NF membranes created from advanced materials are becoming increasingly popular due to their ability to depollute wastewater in a variety of circumstances. Tailoring the NF membrane’s properties to efficiently remove heavy metal ions from wastewater, interfacial polymerization, and grafting techniques, along with the addition of nano-fillers, have proven to be the most effective modification methods. This paper presents a review of the modification processes and NF membrane performances for the removal of heavy metals from wastewater, as well as the application of these membranes for heavy metal ion wastewater treatment. Very high treatment efficiencies, such as 99.90%, have been achieved using membranes composed of polyvinyl amine (PVAM) and glutaraldehyde (GA) for Cr^3+^ removal from wastewater. However, nanofiltration membranes have certain drawbacks, such as fouling of the NF membrane. Repeated cleaning of the membrane influences its lifetime.

## 1. Introduction

Surface and groundwater resources are at risk of degradation and pollution due to heavy metal ions present in discharges from various industries. The harmful effects of heavy metal ions on human health require their complete removal from various wastewaters using advanced treatment technologies [1]. The manufacturing processes of various goods, such as paints, vehicle batteries, pigments, and fertilizers, mainly lead to the pollution of water supplies [2]. Overexposure to heavy metal ions can cause severe diseases to humans and animals [3]. Table 1 shows some of the heavy metals present in wastewater, their effects on human health [4], and their concentrations permitted in wastewater according to NTPA 001/2002 [5]. Conventional methods for wastewater treatment polluted with heavy metal ions include electrochemical treatment, flotation, ion exchange, and chemical precipitation [6,7,8]. Each method has its own restrictions, such as the formation of a huge amount of sludge in the chemical precipitation approach [9,10], low treatment efficiency, high resin cost, and problems in regenerating spent resin in the ion exchange process [11,12].

Currently, membrane-based technology is considered an effective and scalable method for removing heavy metal ions present in wastewater [13]. The most commonly used membrane materials are polymeric, ceramic, and hybrid substances [14], but the use of polymeric membranes is the preferred choice due to their ease of operation, excellent selectivity rates, and surface modification [15]. Microfiltration (MF), nanofiltration (NF), ultrafiltration (UF), and reverse osmosis (RO) are the four classifications of membranes used for membrane-based technologies [16]. However, RO and NF have become the most suitable technologies for water treatment and desalination [17,18,19].

**Figure 1:**
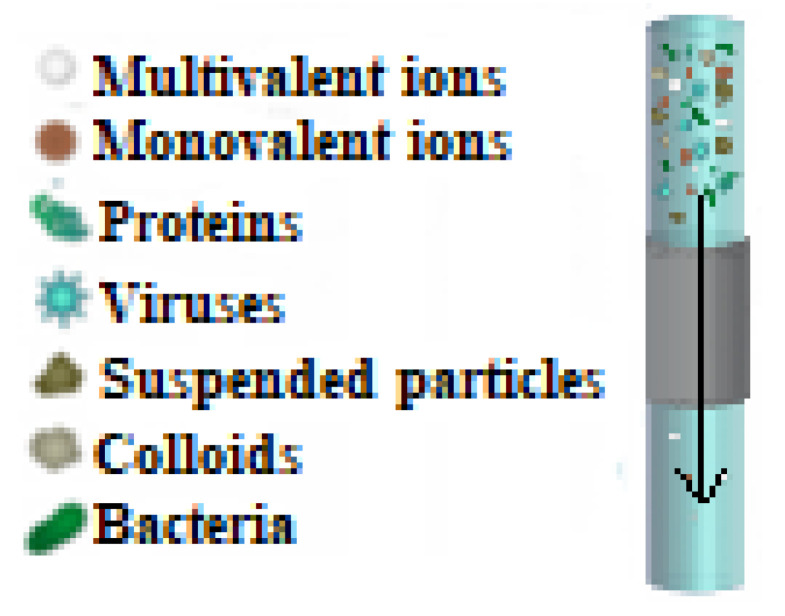
Principle of the method for pollutants removal from wastewater using nanofiltration membrane.

Nanofiltration (NF) is a membrane-based separation technique that utilizes hydrostatic pressure to transport molecules across semipermeable membranes (Figure 1). This method allows low-molecular-weight solutes and solvents to move through the membrane while larger molecules are trapped. NF membranes have a molecular weight threshold of around 400–500 Da, have pore diameters between 0.5 and 2 nm, and require working pressures in the range of 10–50 bar. They have the capacity of retaining neutral species with a molecular weight between 200 and 300 g/mol and reject inorganic ions through a combination of electrostatic interactions between the charged membrane and the ions and size exclusion [20,21]. NF provides several advantages over other membrane technologies, such as stronger rejection of higher flux and divalent ions, reduced energy usage, and lower operating pressure. This makes it a promising technology for removing oil and grease, suspended particles, heavy metals, dyes, and other chemicals from industrial effluents and drinking water [22,23]. NF membranes can be improved by modifying their composition, morphology, and structure to enhance their permeability, selectivity, and chemical and mechanical stability. Thin film composite (TFC) membranes are commercially used due to their heavy metal removal rate, high water permeability, and strong mechanical and chemical stabilities [24,25,26].

Membrane desalination is a highly efficient technique for treating saline water and wastewater and has received significant attention recently. The key challenge in membrane research is to fabricate highly permeable and stable membranes with excellent selectivity, favorable physico-chemical properties, and antifouling properties [27,28,29]. To achieve this objective, nanotechnology has appeared as a promising approach for the development of new membranes for industrial applications. The synthesis of nanoparticles with strong sorption of pollutants, high compatibility with membrane matrix, and a high specific surface is the most important. Additionally, the size of particles is a critical factor that affects the membrane’s mass transfer and separation performance, as well as the potential for their reuse [30,31,32,33].

Although nanomaterials, particularly inorganic fillers, can provide excellent permeation pathways to active sites for the pollutant’s adsorption, unwanted defects between the polymer matrix and the fillers, weak nanomaterial dispersion on the membrane surface or in the membrane structure, and nanomaterial agglomeration can greatly reduce the selective separation of pollutants [30].

This article focuses on nanofiltration membranes and their use for the removal of heavy metals from wastewater. However, many membranes have been synthesized and studied for the removal of dyes [34,35,36], fluorides [37,38,39], and others. Some examples are given in the following paragraphs and Table 2.

Metal-organic framework materials (MOFs) having an ultra-high specific surface area were studied for the degradation of Congo red dye from wastewater. The researchers developed ZIF-67@wood composites by growing ZIF-67 onto the wood surfaces. Additionally, hydrophilic magnetic WC-Co composites were synthesized by carbonizing ZIF-67@wood. These WC-Co composites effectively combine the magnetic core-shell Co/C nanoparticle active sites with the carbonized wood’s hierarchical porous structure. At dye concentration of 1200 mg/L, the Co/C-1000 exhibits a remarkable removal efficiency of 99.98% under gravity. When connected to a peristaltic pump with a flux of 1.0 × 10^4^ L m^−2^h^−1^ for a congo red solution (100 mg/L), the Co/C-1000 filter demonstrates an impressive removal efficiency of nearly 99.28%. Furthermore, the Co/C-1000 filter offers high reusability. The adsorbed dyes can be easily eliminated through simple burning [34].

Researchers have investigated a novel macroporous membrane based on Metal-Organic Frameworks (MOFs) for highly efficient uranium extraction from seawater through continuous filtration. To achieve this, UiO-66 was modified with poly (amidoxime) (PAO) to enhance its dispersion in a solution containing graphene oxide and cotton fibers in N,N-dimethylformamide. The resulting MOF-based macroporous membrane, which exhibits superhydrophilicity, was easily fabricated by simple suction filtration. The membrane demonstrated a uranium extraction capacity of 579 mg·g^−1^ in simulated seawater containing 32 ppm of U after only 24 h. Notably, the 100 mg of UiO-66@PAO membrane effectively removed 80.60% of uranyl ions from seawater [40].

Magnetically separable electrospun nanofibers composed of La-Mn-Fe tri-metal oxide (LMF NFs) for fluoride removal were synthesized. The LMF NFs were prepared through electrospinning followed by heat treatment. Notably, the nanofibers exhibited a uniform distribution of magnetic Fe_3_O_4_ nanoparticles along their axis, effectively preventing agglomeration. The magnetic nature of the fibrous LMF NFs facilitated their easy separation from the solution by applying an external magnet after fluoride adsorption. The highest efficiency for fluoride remediation was 99.33% [39].

**Figure 2:**
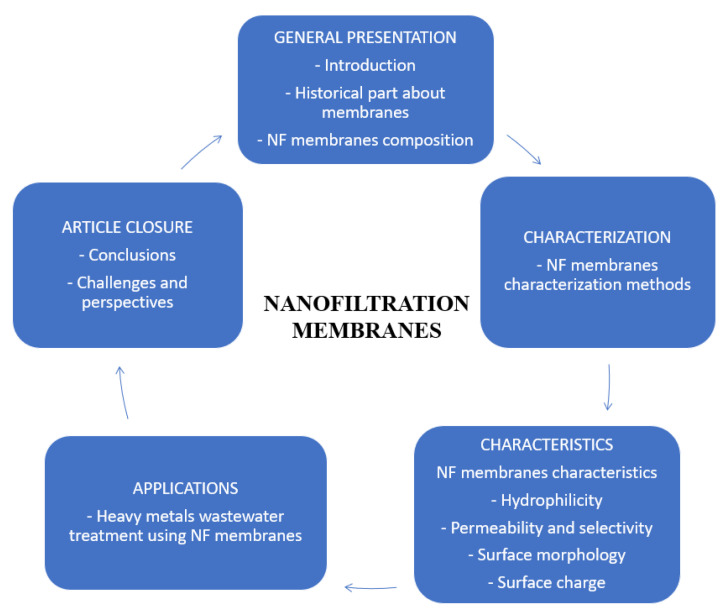
The information about NF membranes presented in this review.

Further, the nanofiltration membranes and the characterization methods and characteristics of NF membranes are described. Finally, this study presents the applications of NF membranes for wastewater treatment containing heavy metal ions. A representative scheme of the article composition is presented in Figure 2.

## 2. Nanofiltration Membranes

**Figure 3:**
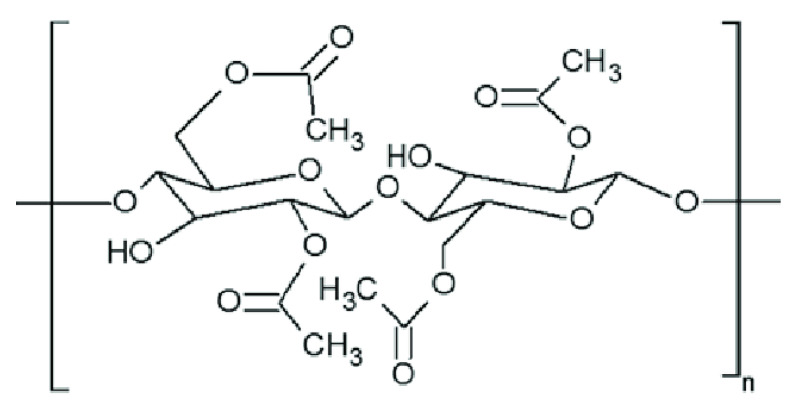
Cellulose acetate chemical structure.

**Figure 4:**
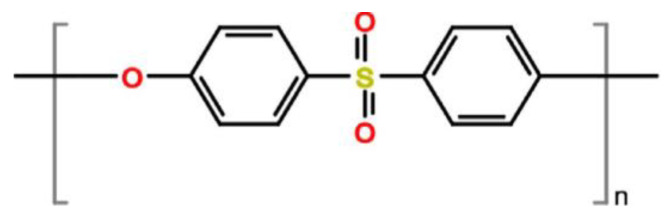
Polyether sulfone chemical structure.

**Figure 5:**
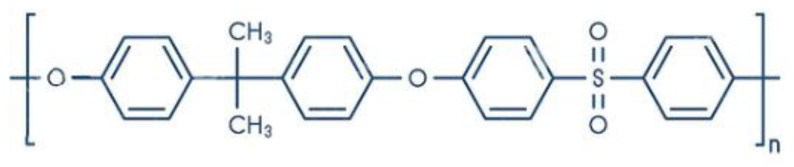
Polysulfone chemical structure.

**Figure 6:**
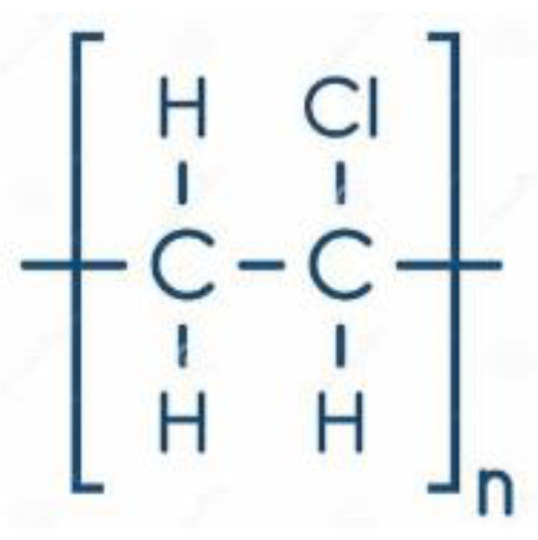
Polyvinyl chloride chemical structure.

**Figure 7:**
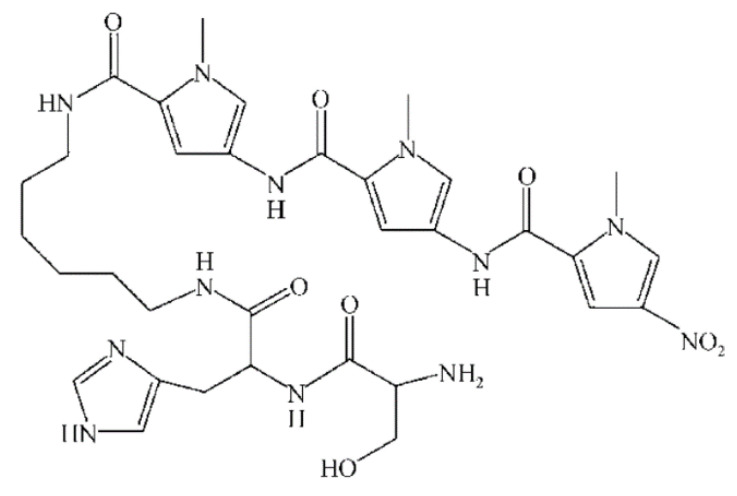
Polyamide chemical structure.

**Figure 8:**
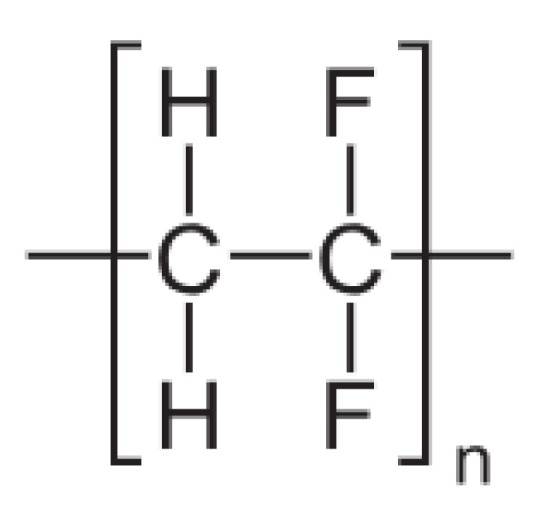
Polyvinylidene fluoride chemical structure.

Initially, NF was developed as an offshoot of RO and UF; hence, it was initially called open RO or tight UF, depending on its application. The obtaining of Loeb-Sourirajan (L-S) asymmetric or anisotropic cellulose acetate membranes in the late 1950s for seawater desalination provided the foundation for the development of NF membranes, as well as pressure-driven membranes in the RO and UF sectors in the early 1960s [41]. These membranes served as the basis for the development of today’s membranes in the UF and RO sectors. Lately, an asymmetric UF was developed, supported by RO composites with a submicron coating on a selective layer. Advances in RO and UF technologies led to the emergence of a new field known as nanofiltration (NF), which was researched and developed for approximately 15 years beginning in 1960. In the 1970s, a range of cellulose acetate asymmetric membranes covering the whole spectrum from RO to UF were available [42]. Limitations of cellulose acetate as a membrane material were observed, and thus NF could not be widely applied [43]. Thus, cellulose acetate (Figure 3) has been replaced by other materials such as polyether sulfone (PES) (Figure 4), polysulfone (PSU) (Figure 5), chlorinated polyvinyl chloride (PVC) (Figure 6), polyamide (PA) (Figure 7), or polyvinylidene fluoride (PVDF) (Figure 8). Polymers, such as PVC, can be leached into the treated water due to continuous exposure to high pressure [44]. Even so, the NF membranes were not good enough to achieve the required selectivity/flux balance [45,46]. Composite membranes based on interfacial polymerization (IP) of UF supports having a submicron selective barrier were then developed [47]. Another alternative was the development of ceramic and inorganic NF membranes [48].

**Figure 9:**
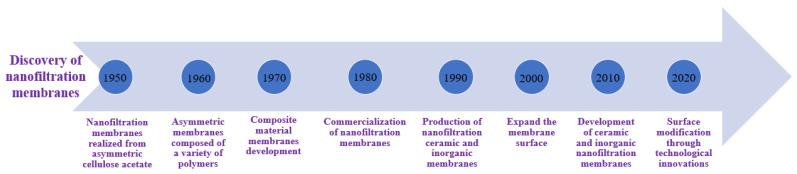
Timeline representing the use of membranes over time since their discovery in 1950.

NF membranes, which are commonly composed of three TFC layers, have a support layer on top that facilitates mass transportation. The second layer acts as a UF or MF membrane and supports the first layer. By this mechanism, the third active layer support layer controls the hydrophilicity, membrane charge, and surface features. Regenerated cellulose and polyvinyl alcohol are two common hydrophilic materials used to manufacture NF membranes [49], but other synthetic polymers have also gained popularity since the 2010s due to their suitability for specific applications [50]. However, membrane technology has some drawbacks, such as membrane fouling and high initial investment costs, which necessitate additional treatment procedures [4]. A major problem is also the membranes’ biofouling with bacteria and soluble microbial products. Biofouling can create significant problems in terms of removal efficiency during filtration and flux [51]. To enhance the performance of NF membranes, various methods such as plasma and chemical treatment, UV radiation, additive blending, grafting, crosslinking, and adsorbed coatings are used to modify the membrane surface. Cross-linking with hydroxyl compounds, for example, is used to improve membrane stability, increase the hydrophilicity of PA surfaces, and decrease molecular cut-off [52]. Recently, the development of positively charged NF membranes to remove heavy metals using the IP approach has attracted significant attention. Studies have demonstrated that the addition of nanoparticles and the creation of interlayers and surfactants can enhance the permeation flux of NF membranes [53]. The timeline for the discovery and use of nanofiltration membranes is shown in Figure 9.

Commercially available NF membranes are known for their pore size of approximately 1 nm. They have a molecular weight cutoff between 300 and 500 Da. While NF membranes exhibit low salt rejection (10–30%) for monovalent salts (e.g., NaCl), they exhibit high salt rejection (80–100%) for divalent salts (e.g., Na_2_SO_4_). These inherent characteristics distinguish NF membranes from RO membranes, giving NF membranes superior selectivity for various classes of ions and small molecules. As a result, NF membranes find extensive utility in specialized applications in various industries, including water and wastewater treatment, biotechnology, food engineering, and pharmaceuticals [54].

## 3. Characterization Methods of NF Membranes

Recent studies have focused on developing membranes that can simultaneously increase rejection rates and solute permeation rates. To achieve this, a thorough understanding of various membrane parameters is essential, and various characterization techniques can aid in this process. Thus, before conducting nanofiltration experiments, it is beneficial to characterize the NF membranes using different methods to determine their physical and chemical properties, stability, and separation performance. There are various analytical instruments available that can be used to characterize NF membranes, including several chemical and physical methods that determine pore size or nanopore distribution on the surface, surface roughness, surface morphology, compatibility, topography, and interactions between membrane and nanoparticles [55]. Fourier transform infrared spectroscopy (FTIR) can detect the PA layer band and the substrate band, elucidating membrane composition, morphology, and structure because the IR beam depth exceeds the PA layer thickness [56,57]. Zeta potential is a commonly used characterization method to establish the surface charge property of NF membranes in an aqueous environment at different pH levels [58]. The zeta potential analysis is important to understand the acid-base properties, separation efficiency, and fouling tendency of the NF membrane under different pH conditions. Electro-osmosis can be used to measure the zeta potential of the membrane pore perpendicular to the membrane surface [59]. X-ray photoelectron spectroscopy (XPS) is a spectroscopic method that provides information regarding the basic composition of NF membranes and the cross-linking structure of the PA layer, which is useful for research purposes. The X-ray diffraction (XRD) method is helpful in determining the NF membrane’s crystalline properties, including the nanoparticle incorporation on the membrane surface [55]. Additionally, nuclear magnetic resonance (NMR) is a useful method to characterize the freshly prepared monomer organic structure and any change in the membrane surface cross-linking structure [60]. Numerous instruments and methods are available to estimate the NF membrane’s physical properties, making research in this area intriguing. The gas adsorption-desorption technique, also known as the Brunauer-Emmett-Teller (BET) method, is one such method that provides a direct assessment of pore size distribution [61]. Composite membrane morphology analysis, from nanometers to hundreds of micrometers, can be accomplished using various electron microscopy methods. Three commonly used types of electron microscopy for investigating the morphological properties of NF membranes are field emission scanning electron microscopy (FESEM) [56,62], scanning electron microscopy (SEM) [55], and transmission electron microscopy (TEM) [63]. These methods offer the advantage of producing visual data on the morphology of the membrane at a desired resolution. TEM can directly determine pore size and distribution by using reverse surface impregnation [63]. SEM can be used to examine the membrane surface, membrane cross-section, fouling layers, and thickness [55]. Positron annihilation spectroscopy (PAS) is an advanced tool used to analyze molecular pores and vacancies in membrane materials in a non-destructive and descriptive-analytical way [64]. Atomic force microscopy (AFM) can directly determine surface roughness, topography, pore size distribution, and force interactions between the membrane and colloids [65,66]. The hydrophilicity, hydrophobicity, or wettability of the NF membrane can be determined with the help of a contact angle analyzer [67].

Solute-solute rejection selectivity is a crucial aspect of membrane performance, as it determines the membrane’s ability to selectively reject different solutes in water. This selectivity relies on various rejection mechanisms, including steric hindrance, the Donnan effect, and the dielectric effect [68,69,70,71]. A key membrane property that governs the solute-solute rejection selectivity is represented by the distribution of the membrane pore size [72,73,74]. A more uniform pore size distribution is considered to contribute to a higher selectivity of solute-solute rejection [75].

Usually, the membrane pore size distribution is determined by fitting the model to experimentally acquired rejection data for a range of different-sized probe solutes [76,77]. Conventionally, NF membrane pores are supposed to follow a log-normal distribution, characterized by two adjustable parameters representing non-uniformity and the median pore size. A method to obtain the pore size distribution of the membrane is by equating the cumulative distribution function to the solute rejection profile as a function of size [78]. The issues with this commonly used traditional approach stems from its underlying and unreasonable assumptions. These assumptions suggest that pore rejection is a binary function, either 0 or 1, solely based on pore size, and that the water flux that passes through pores is unaffected by pore size. To achieve a more accurate representation of membrane pore size distribution, it is necessary to consider the intricate effects of pore size on solvent flux and solute rejection [79].

Several mathematical models have been created to establish a connection between membrane properties and membrane performance [80,81,82]. Among these, a notable one is the model Donnan steric pore. This model characterizes water flux in relation to pore size using the Hagen–Poiseuille equation while also assessing solute flux in relation to pore size by considering the steric and electrostatic influences on solute partitioning at the interface and subsequent mass transport within the pores, as described by the extended equation of Nernst–Planck [83].

## 4. NF Membranes Characteristics

### 4.1. Hydrophilicity

To evaluate a membrane’s hydrophilicity, the water contact angle is utilized. Increasing a membrane’s hydrophilicity can enhance its permeability and antifouling properties. Although most solids possess natural roughness, this roughness is usually not enough to maintain a superhydrophilic state on the material’s surface. In theory, any natural or artificial substance can be chemically processed or mechanically roughened to create a super hydrophilic surface, or it can be broken down into sub-microscopic particles and stored to create a super hydrophilic coating. Titanium dioxide (TiO_2_) and zinc oxide (ZnO) are two inorganic substances that are commonly used because of their photoinduced self-cleaning capacities [84]. Silicon dioxide (SiO_2_) is extensively studied for its low cost and hydrophilicity. Various processes, such as electron beam, X-ray or ion surface irradiation, microwave treatment, and plasma, can be used to modify the surface chemistry of a polymer and increase its hydrophilicity. In order for a polymer to become super hydrophilic, the treatment must affect surface roughness or be applied simultaneously with surface roughening [85]. Increasing the surface roughness will decrease the strength of the NF membrane due to the thinning of the outer separation layer and, at the same time, the NF membrane [86]. Various coating techniques have been employed to modify the surface wetting, including dip coating, sol-gel, thermal, layer-by-layer assembly, electrospinning, electrodeposition, ion beam irradiation, femtosecond laser irradiation, spin coating, plasma irradiation, chemical vapor deposition, and spray coating. The production of super hydrophilic surfaces typically involves the use of low surface energy materials, surface roughness, or a combination of both. The membrane’s performance depends on properties such as surface energy, pore size, and wettability [85].

In recent years, interest has been growing in coatings that exhibit switchable wetting properties. Some coatings have been developed that can transition between superhydrophobic and superhydrophilic states, such as those produced using the sol-gel technique [87]. Graft polymerization has emerged as a practical alternative for improving the polymeric membranes’ hydrophilicity and enhancing their antifouling properties [88]. This technique involves attaching hydrophilic polymer chains to the surface of the membrane and offers advantages such as long-term hydrophilicity maintenance and high water flux [85].

### 4.2. Permeability and Selectivity

The energy consumption and effectiveness of NF processes are determined by the selectivity of NF membranes, which is largely influenced by pore size distribution and Donnan effects. A narrow pore-size distribution can play a crucial role in obtaining high efficiency of selective solute separation, while the steric resistance and charge interactions of NF membranes determine their selective properties. Manipulating the membrane surface charge can increase selectivity, specifically for charged solutes. Membrane fouling, which causes the membrane pore diameters to become smaller, can decrease the water flux through NF membranes during operation. Heavy metals can have a significant impact on membrane fouling by altering sludge properties or causing inorganic fouling [89]. Inorganic fouling by heavy metal compounds can be permanent, requiring cleaning with acids like citric acid [90]. Researchers have made various attempts to improve membrane selectivity by creating NF membranes with uniform pore diameters or modifying the charges on or in the selective layer or hydrophilicity, with some success in minimizing the fouling of membranes [91]. Increasing the selectivity of NF membranes can lead to improved membrane permeance while maintaining high rejection of ions/molecules. One approach for enhancing NF membrane selectivity is by incorporating nanofillers into the polymer matrix, creating new molecular transport channels. Metal-organic frameworks and covalent organic frameworks are promising materials for increasing membrane selectivity due to their high surface area, controllable pore structure, strong thermochemical stability, and functionalized pore walls. Metal-organic frameworks and covalent organic frameworks can tailor their pore shape and size and chemical design versatility through post-functionalization or by combining their ligands. Additionally, their cavities can be customized for specific applications and can facilitate beneficial interactions with polymers [92].

### 4.3. Surface Morphology

The effectiveness of membrane filtration in heavy metal removal depends heavily on the surface coating and morphology of the membrane, as they can impact both fouling and anti-fouling performance. The surface topography of a membrane, which includes its roughness, lay, waviness, and flow, can be affected by a variety of factors such as vibrations, manufacturing processes, work deflections, stresses, and the material’s internal structure [93].

The effect of surface roughness on membrane performance remains a significant challenge. It was observed that a commercial thin film composite membrane fouled more quickly than a cellulose acetate membrane, attributing the effect to the thin film composite membrane’s rougher surface [94]. It was demonstrated that, for certain NF membranes, fouling is closely related to surface roughness [95], as colloidal particles tend to accumulate in the valleys of a rough membrane surface due to increased interactions between them. This obstructs the valleys and leads to increased fouling on rougher surfaces [96]. Thus, the effect of surface roughness on membrane performance remains a complex issue. However, different results have been observed for organic particles [97,98]. Adhesive forces, which refer to the interaction between the membrane surface and the organic particles, are thought to be crucial factors in fouling [99]. In the fouling process, particles directly contact the membrane surface, and the interaction between the membrane surface and the organic particles determines the extent of fouling. On the other hand, after the formation of the gel layer, the interaction between organic particles becomes very important. It is plausible that a smoother surface would present less adsorption for the organic molecules, while a more heterogeneous and rougher surface would have a greater surface area and be more effective at adsorption [100].

Chemical surface modification is a technique used to decrease surface roughness by allowing chemicals in the liquid phase to enter pores more effectively, providing a smoother surface [101]. Atomic layer deposition is an alternative method used to manage roughness. It is a process of self-limiting surface reaction that produces uniform thin coatings with flawless intactness and an atomic-scale-controllable thickness [102]. By adjusting the total number of atomic layer deposition cycles, the thickness of the thin film can be precisely controlled at the atomic scale during deposition [103,104]. Surface hydrophilicity can be achieved using atomic layer deposition of alumina [105,106]. TFC-PA (thin film composite—polyamide) membranes were treated with atomic layer deposition to enhance their hydrophilicity and anti-fouling ability [107]. Additionally, low-temperature plasma discharges offer a flexible and controllable method for homogenous surface treatments, allowing for a wide range of conceivable surface functionality and minimizing damage [108].

### 4.4. Surface Charge

NF membranes can acquire an electric charge through various mechanisms when they come in contact with an aqueous electrolyte solution. For example, potential mechanisms include the adsorption of ions from solutions, ionic surfactants, and charged macromolecules on surfaces; the adsorption of polyelectrolytes; and the separation of functional groups [59,109,110,111]. This process can occur on the membrane’s external surface as well as its internal pore surface. Because the system must maintain electroneutrality, the distribution of ions is affected by surface charges. Thus, the surface becomes charged, leading to the development of an electrical double layer and the neutralization of excess counterions present in the surrounding solution. In alkaline or neutral settings, NF membranes tend to be negatively charged, and in highly acidic settings, positively charged. The surface charge of the NF membrane is helpful for selectively intercepting multivalent ions. Due to the set negative charge of the polymer backbone (which usually contains sulfonic acid and carboxylic acid), commercially available NF membranes typically have a negative charge [59,109,110,111,112,113,114].

Negatively charged NF membranes have been found to have better retention for divalent or multivalent anions having the same charge as the membrane surface due to the steric hindrance and Donnan effect [115]. The retention of heavy metal cations is poor in commercially available NF membranes, with rejection rates reported to be as low as 12% for PbCl_2_ and up to 90% for CdCl_2_, depending on the membrane and conditions used [116]. A lower pH in the feed solution relative to the isoelectric point of the membrane can improve the retention of heavier metal cations. The selectivity of the membrane improves with greater charge density, and a positively charged surface on an NF membrane can facilitate the retention of divalent or multivalent cations because of electrostatic repulsion [117].

Three main methods have been developed for producing positively charged NF membranes, namely phase inversion, interfacial polymerization, and surface modification (which includes surface grafting, surface deposition, and surface cross-linking) [118]. Producing composite membranes that are positively charged using surface modification and interfacial polymerization often necessitates the use of harmful or carcinogenic chemicals, as well as many preparation stages [119]. As an alternative, integrally skinned asymmetric membranes can be created using a simpler cross-linking approach and phase inversion methodology [120]. By introducing nitrogen groups or quaternary amines to the surface and interior pores of the membrane, it is reported that positively charging the integrally skinned asymmetric membrane can be possible [121].

## 5. Applications of NF Membranes for Heavy Metals Wastewater Treatment

**Figure 10:**
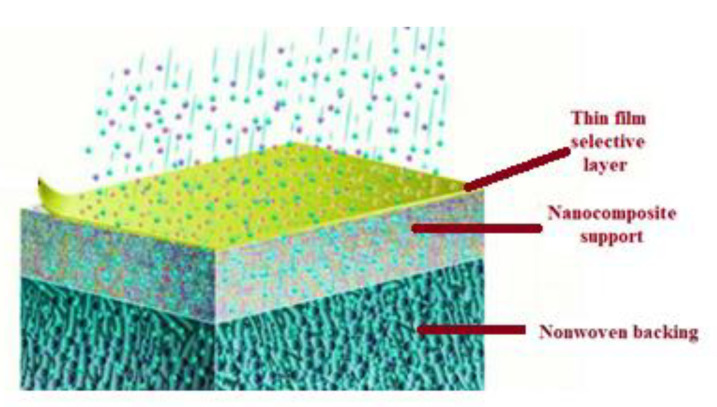
Nanofiltration membrane.

NF membranes (Figure 10) have been recognized and approved worldwide for their remarkable durability, low energy consumption, affordability, and a heightened capacity to remove pollutants. Choosing NF for membrane separation processes not only ensures cost-effectiveness but also promotes environmental friendliness. New thin film composite (TFC) and thin film nanocomposite (TFN) membranes have been developed using a vapor-phase interfacial polymerization process. These membranes were designed to remove heavy metal ions [122]. Some researchers utilized triethanolamine as a crosslinking agent to create the nanofiltration membranes polyethyleneimine/trimesoyl chloride (PEI/TMC) for studying the removal of heavy metal ions from wastewater. The presence of lone pair electrons on triethanolamine’s nitrogen atoms increased the positive charges and reduced the pore sizes, leading to a significant increase in the rejection rate of heavy metal ions in polluted water. The calculated rejection percentages were approximately 97.00% for Ni^2+^, Cu^2+^, Zn^2+^, Cd^2+^, and 92.00% for Pb^2+^. The modification of the membrane with triethanolamine also enhanced its hydrophilicity, resulting in a flow increase of 13.6 Lm^−2^h^−1^bar^−1^. These improvements make the triethanolamine-modified membrane highly suitable for industrial applications in the removal of divalent heavy metal ions from wastewater, offering superior performance and stability [123].

Graphene-based membranes, which are two-dimensional nanofiltration membranes, have gained prominence as a successful separation technique due to their distinctive bounded channels [124]. An NF membrane was developed through the use of reduced graphene oxide (rGO) using a plasma-assisted in-situ photocatalytic reduction technique. Initially, graphene oxide-silver (GO-Ag) composite sheets were formed and collected on the membrane surface using vacuum filtration. Subsequently, the GO-Ag layer was in situ reduced into rGO-based membranes by employing a plasmonic photocatalyst, namely Ag nanoparticles. The modification from GO-Ag to rGO-Ag resulted in enhanced water flux, stability, and rejection capacity of the membrane when exposed to toxic heavy metal ions (such as Cr(III), Cr(IV), Pb(II), and Cu(II)) solutions. The experimental results indicated the potential of the prepared membrane to effectively separate complex wastewater systems containing mixed solutions of Cr(IV) and Cr (III) [125].

A highly positively charged NF selective layer has been successfully developed on the hollow fiber membrane outer surface using iodomethane quaternization and surface grafting techniques. Comparative studies were conducted to evaluate the effectiveness of the membrane that was prepared compared to other membranes with single charges. The resulting membrane composed of polyvinyl amine (PVAM) and glutaraldehyde (GA) exhibited exceptional removal capacity, with rejection rates of 99.40, 99.60, and 99.90% for the heavy metal ions Ni^2+^, Cu^2+^, and Cr^3+^. Moreover, it demonstrated a higher permeate flux of approximately 27.9 Lm^−2^h^−1^bar^−1^. The NF membrane also showed favorable antifouling properties against heavy metal ions. The application of quaternization and surface grafting significantly enhanced the NF membrane’s performance, making it a promising advancement in the field of membrane separation [126]. The tubular AFC 40 nanofiltration membrane’s performance was investigated using real wastewater samples from the industry. The results of the experiment indicated that the membrane was effective in separating zinc, as it exhibited high rejection rates and a significant permeate flow. The rejection percentage exceeded 99.00% in most cases, except for the lowest pressure and lowest concentration. The rejection rate varied depending on the feed flow rate. The highest rejection rate of 98.66% was achieved at a flow rate of 9 Lmin^−1^ and a pressure of 10 bar. Notably, the pH value of 3 yielded the highest rejection rate, reaching 99.29%. This study confirmed that the membrane AFC 40 is well adapted for efficiently removing zinc from wastewater [127].

A novel and controllable approach is introduced for the synthesis of Fe_3_O_4_@PDA-g-L-Cys materials. Initially, Fe_3_O_4_ nanoparticles were prepared and then coated with PDA through auto polymerization of dopamine monomer, resulting in the formation of Fe_3_O_4_@PDA core-shell nanoparticles. Subsequently, the PDA-coated Fe_3_O_4_ surface was modified by grafting L-cysteine and introducing amine and carboxyl groups. The core/shell composites that resulted can be conveniently recycled through magnet separation. These core-shell nanomaterials were used for the efficient removal of lead ions from wastewater. The Fe_3_O_4_@PDA and Fe_3_O_4_@PDA-g-L-Cys materials demonstrate maximum adsorption capacities of 31.84 mg/g and 46.95 mg/g [128].

Table 3 presents some examples of NF membranes that have been studied for the removal of heavy metal ions from wastewater.

In contrast, wastewater often contains complex substances that must be separated to protect the well-being of organisms in both land and water environments. A study on the depollution of industrial wastewater that contained chloride-rich effluent was conducted. The wastewater was collected from a clarified tank in India. For the experiment, the research team developed two composite NF membranes using polyethylene glycol, polysulfone, and zinc chloride. Prior to utilizing the NF membrane for wastewater treatment, a pretreatment process involving granular activated carbon (GAC) was employed. Under optimal conditions of 1390 kPa pressure and a crossflow rate of 80 L/h, the rejection percentages were as follows: 32.00% for fluoride, 27.00% for nitrate, and 70.00% for phosphate at an effluent pH of 7.95. NF membrane in-situ washing with tap water resulted in a permeate flux recovery of up to 97.00%. The impact of polarization on system performance was assessed by applying resistance to the series model. The experiment’s findings indicate the potential success of scaling up this system in a spiral-wound configuration [129].

## 6. Conclusions

Heavy metals such as As, Cd, Cr, Cu, Ni, Zn, Pb, Hg, and Ag pose significant risks when they are present in wastewater. Various researchers have conducted numerous experiments utilizing conventional wastewater treatment techniques to eliminate these heavy metals from wastewater. Despite the establishment of techniques such as electrochemical, adsorption, reverse osmosis, nanofiltration, ultrafiltration, or microfiltration, there is currently no comprehensive review addressing the NF membrane modification for heavy metal removal. This review article provides valuable insights into NF membranes, including their preparation, advancements, and applications. Several challenges and limitations must be overcome for these membranes to have a substantial impact on wastewater treatment processes.

A noteworthy finding is that the careful integration of organic, inorganic, or hybrid nanofillers in polymer membranes can lead to a high removal percentage for heavy metal ions from wastewater. Among the introduced NF membranes, the B-Cur/PES (Curcumin/Polyethersulfones), Ti_3_C_2_TX/EDA (Titanium Carbide/Ethylenediamine), and GO-PAMAM/PES (Graphene Oxide-Poly amidoamine/Polyethersulfones) composite-based membranes, which incorporate organic, inorganic, or hybrid nanofillers, demonstrate higher potential compared to others. These membranes, respectively, exhibit heavy metal chelation in polyelectrolytes, a combination of organic and metallic linkers, and an abundance of hydrophilic functional groups. Parameters such as water permeability, membrane fouling, toxicity, reusability, and stability largely depend on the materials employed in membrane fabrication and synthesis methods.

## 7. Challenges and Perspectives

NF membranes are receiving more and more attention and are being studied by researchers for water treatment applications. However, future research should consider improving NF membranes to reduce fouling, and biofouling, or increase their efficiency for industrial use. Some ideas for improving NF membranes are: improvement of certain technical characteristics of NF membranes to mitigate fouling, increase durability, and improve stability; in order to control the membrane’s biofouling, it will be necessary to understand the physicochemical interactions between bacteria and the membrane and between the membrane and the soluble microbial product; in order to commercialize NF membranes more easily, the reduction of energy costs for nanofiltration systems should be considered; improving the technology to be able to detect fouling in time and take the necessary measures. The potential capacity of NF membranes to remove heavy metal ions (e.g., Mn^2+^, Zn^2+^, Co^2+^, Cu^2+^, Ni^2+^, Pb^2+^, Cd^2+^, and others) from wastewater can be improved by surface modification through interfacial polymerization and grafting, appropriate systematic membrane synthesis, and modification of membrane structure by the addition of nanofillers. Fouling of NF membranes directly influences their chemical resistance and lifetime. For the most efficient process, energy consumption, choice of materials, operating conditions, cleaning chemicals, and overall environmental impact must be considered. For wastewater applications, a major drawback is the frequency of membrane cleaning, which influences the lifetime of the membranes and requires a well-thought-out strategy to prevent rapid fouling of the membranes.

## Figures and Tables

**Table 1:** Main organs and systems of humans affected by heavy metal ions present in wastewater and recommended water load limit values of NTPA 001/2002 [5].

Heavy Metals	Effects upon the Main Organs and Systems	Permitted Concentration [mg/dm^3^]
Mercury (Hg^2+^)	Reproductive system, cardiovascular system and immunological system, kidneys, liver, brain, lungs	0.05
Chromium (Cr) Cr^3+^/Cr^3+^+Cr^6+^/Cr^6+^	Gastrointestinal system and reproductive system, taste, brain, pancreas, kidneys, liver, skin, lungs	-/1.00/0.10
Cadmium (Cd^2+^)	immunological system and cardiovascular system, brain, kidneys, lungs, bones, liver	0.20
Zinc (Zn^2+^)	Skin, stomach	0.50
Arsenic (As^+^)	Immunological system, endocrine system, metabolic system and cardiovascular system, brain, kidneys, skin, lungs	0.10
Nickel (Ni^2+^)	Gastrointestinal system, skin, kidneys, and lungs	0.50
Copper (Cu^2+^)	Immunological system, haematological system, and gastrointestinal system, lungs, kidneys, cornea, liver, brain	0.10
Manganese (Mn^2+^)	Respiratory tract, brain.	1.00
Lead (Pb^2+^)	Cardiovascular system, reproductive system immunological system, and haematological system, lungs, spleen, kidneys, brain, bones, liver	0.20

**Table 2:** Membranes synthesized and used for the removal of various pollutants.

Membranes	Pollutants	Treatment Efficiencies [%]	Ref.
ZIF-67@wood composites	congo red dye	99.28	[34]
MOF-based macroporous membrane	uranium	80.60	[40]
La-Mn-Fe tri-metal oxide	fluoride	99.33	[39]

**Table 3:** Examples of membranes for the removal of heavy metal ions from wastewater.

NF Membranes	Heavy Metal Ions	Removal Efficiencies (%)	Ref.
PEI/TMC	Ni^2+^, Cu^2+^, Zn^2+^, Cd^2+^	~97.00	[123]
	Pb^2+^	92.00	
PVAM/GA	Ni^2+^	99.40	[126]
	Cu^2+^	99.60	
	Cr^3+^	99.90	
AFC 40	Zn^2+^	99.29	[127]

## Data Availability

Not applicable.
